# GM-CSF Dependent Differential Control of *Mycobacterium tuberculosis* Infection in Human and Mouse Macrophages: Is Macrophage Source of GM-CSF Critical to Tuberculosis Immunity?

**DOI:** 10.3389/fimmu.2020.01599

**Published:** 2020-07-23

**Authors:** Abhishek Mishra, Vipul Kumar Singh, Jeffrey K. Actor, Robert L. Hunter, Chinnaswamy Jagannath, Selvakumar Subbian, Arshad Khan

**Affiliations:** ^1^Department of Pathology and Genomic Medicine, Houston Methodist Research Institute, Houston, TX, United States; ^2^Department of Pathology and Laboratory Medicine, McGovern Medical School, University of Texas Health Sciences Center-Houston, Houston, TX, United States; ^3^Department of Medicine, New Jersey Medical School, Public Health Research Institute, Newark, NJ, United States

**Keywords:** *Mycobacterium tuberculosis*, granulocyte monocyte colony stimulating factor, macrophage, cell death, innate immunity, tuberculosis

## Abstract

Although classically associated with myelopoiesis, granulocyte-macrophage colony-stimulating factor (GM-CSF) is being increasingly recognized for its potential role in innate resistance against tuberculosis (TB). While the GM-CSF is produced by a variety of host cells, including conventional and non-conventional T cells, macrophages, alveolar epithelial cells, the cell population that promotes GM-CSF mediated innate protection against *Mycobacterium tuberculosis* infection remains unclear. This is because studies related to the role of GM-CSF so far have been carried out in murine models of experimental TB, which is inherently susceptible to TB as compared to humans, who exhibit a resolution of infection in majority of cases. We found a significantly higher amount of GM-CSF production by human macrophages, compared to mouse macrophages, after infection with *M. tuberculosis in vitro*. The higher levels of GM-CSF produced by human macrophages were also directly correlated with their increased life span and ability to control *M. tuberculosis* infection. Other evidence from recent studies also support that *M. tuberculosis* infected human macrophages display heterogeneity in their antibacterial capacity, and cells with increased expression of genes involved in GM-CSF signaling pathway can control intracellular *M. tuberculosis* growth more efficiently. Collectively, these emerging evidence indicate that GM-CSF produced by lung resident macrophages could be vital for the host resistance against *M. tuberculosis* infection in humans. Identification of GM-CSF dependent key cellular pathways/processes that mediate intracellular host defense can lay the groundwork for the development of novel host directed therapies against TB as well as other intracellular infections.

Traditionally, it is thought that protective immunity to tuberculosis (TB) is primarily mediated by T cells, with CD4^+^ T cells playing a central role. However, many population-based immunological and genetic studies support the belief that innate immunity is equally important in TB immunopathogenesis (1–3). Macrophages, a critical part of the innate immune system, have paradoxically been also recognized as a primary intracellular niche for the growth and survival of *Mycobacterium tuberculosis* (MTB) (4). While the molecular details of the MTB-macrophage interaction continue to be elucidated, emerging evidence suggest that the phenotypes and functional capacities of the recruited macrophage may play a crucial role in determining the outcome of infection (5). Currently, the elements that govern the tremendous phenotypic heterogeneity and functional plasticity of macrophages are not precisely known. More recent studies indicate that GM-CSF-driven differentiation of monocytes toward a macrophage is critical for its increased responsiveness to microbial pathogens (6–8). The importance of GM-CSF in mediating MTB infection control and inflammation *in vivo* has previously been reported by us and others (9, 10). However, GM-CSF can be produced by a variety of host cells, including conventional and non-conventional T cells, macrophages, and alveolar epithelial cells. Most of the studies that have been conducted in murine models of TB suggest that the production of GM-CSF by lung epithelial cells, conventional, and non-conventional T cells are essential for generating a protective immune response and restricting MTB growth in the lungs (8–11). A recent study, clearly suggested that human macrophages were also able to produce GM-CSF upon infection with MTB, and their antimycobacterial properties correlated with their ability to produce GM-CSF (12). While MTB-infected human macrophages displayed cell-to-cell variability in their antibacterial capacity, cell populations with increased expression of genes involved in the GM-CSF signaling pathway were able to better control MTB growth. The reduced ability of HIV-infected macrophages to produce GM-CSF and control MTB infection further suggests that GM-CSF signaling mediates host defenses.

Murine macrophages are also known to produce GM-CSF in the lung compartment during MTB infection (8). However, in previous studies, it was unclear whether GM-CSF produced by macrophages could also contribute to the protective response against MTB infection in mice, especially during the early phase of infection when conventional T cells (CD4) have not come into effect. It is also important to consider that mice are naturally more susceptible to MTB infection, as compared to humans; therefore, it was intriguing to examine whether differential GM-CSF production occurs in human vs. mouse macrophages.

We thus examined the level of GM-CSF produced by mouse MDMs (monocyte-derived macrophages) compared to human MDMs before and after infection with MTB (Figure 1A). While intrinsic levels of GM-CSF were very low in both human and mouse MDMs, the human MDMs produced relatively higher levels of GM-CSF than did mouse MDMs, even without MTB infection. After infection with MTB, human MDMs produced 2.5- to 5-fold higher levels of GM-CSF compared to mouse MDMs when measured over 7 days. When the ratio of GM-CSF produced by infected and uninfected MDMs was calculated (from Figure 1A), human MDMs (3.02–4.66) were found to have a higher ratio as compared to mouse MDMs (2.62–3.48). This ratio also constantly increased with time in human MDMs whereas it remained more or less stable in mouse MDMs. The comparison of GM-CSF production of infected and uninfected MDMs further indicated that infection with MTB increased the production of GM-CSF more robustly in human macrophages as compared to mouse macrophages. The bacterial burden in mouse MDMs was also significantly higher compared to human MDMs (Figure 1B). Notably, the level of GM-CSF production by human vs. mouse MDMs was different even before infection as well as at the beginning of infection (Day 1) when both of them had similar uptake of the bacilli, which suggests that the differential production of GM-CSF by human vs. mouse MDMs was most likely not driven by their respective bacterial load. We also examined if the induction of GM-CSF production could specifically be related to MTB infection only. Thus, LPS was added to human and mouse MDMs separately before infection with MTB. We, however, and did not find LPS to increase a significant level of GM-CSF production in both human and mouse MDMs (Figure S1) as compared to MTB infection (Figure 1A). This indicated that induction of GM-CSF production by mouse and human macrophages could be driven independent of pattern recognition receptor (TLR4) activation and be more specifically related to MTB infection. Also, when other cytokines produced by MDMs were examined, no significant difference in TNF-α, IL-4, and IL-10 but higher levels of IL-12 production by human MDMs were observed as compared to mouse MDMs after infection with MTB (Figure S2). IL-12 is known to be critical for the maturation and differentiation of antigen presenting cells (13). A positive correlation between GM-CSF and IL-12 production by human MDMs after infection with MTB thus indicates that higher levels of GM-CSF could have driven an alternate maturation, differentiation, and activation of human MDMs as compared to mouse MDMs.

**Figure 1:**
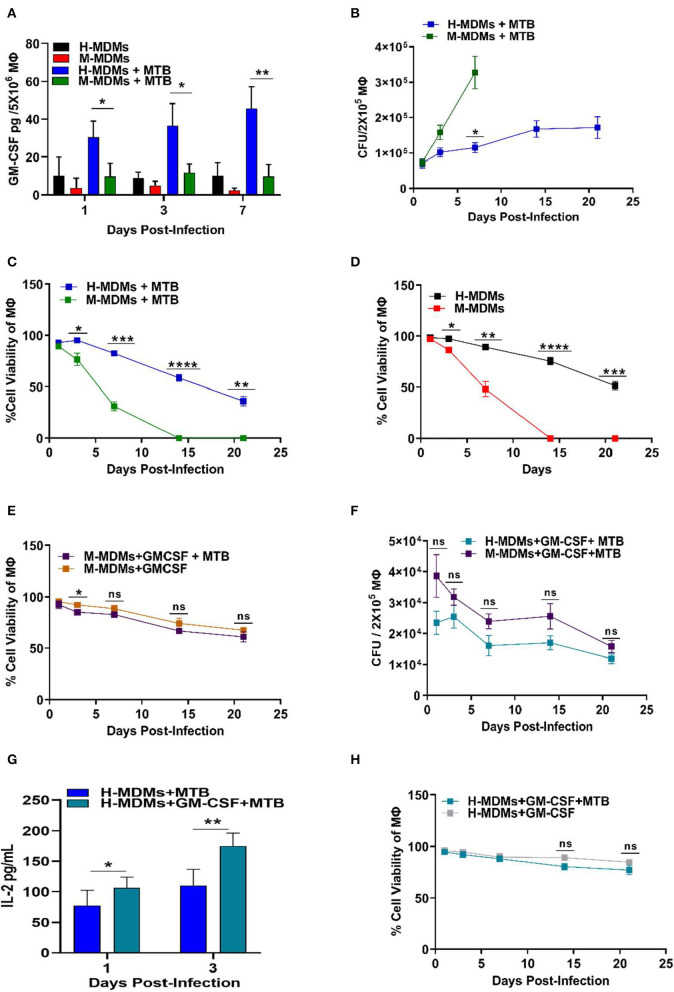
Variability between human and mouse MDMs in their ability to secrete GM-CSF, control MTB infection and cell viability, and present antigens in the presence or absence of exogenous GM-CSF. **(A)** GM-CSF secreted by uninfected and MTB-infected human and mouse MDMs. **(B)** Changes in intracellular bacterial burden of mouse and human MDMs over 21 days. Bacterial burden in mouse MDMs could not be measured after 7 days because of complete death of host cells beyond this time point. **(C)** Cell viability of MTB-infected human and mouse MDMs over 21 days as measured by Alamar blue assay. **(D)** Cell viability of uninfected human and mouse MDMs over 21 days as measured by Alamar blue assay. **(E)** Effect of exogenous addition of GM-CSF on cell viability of MTB-infected and uninfected mouse MDMs with time. **(F)** Changes in bacterial burden with time in human and mouse MDMs after exogenous addition of GM-CSF (2 ng/mL/2 × 10^5^ macrophages). **(G)** Antigen presentation levels (secreted IL-2 levels by MTB Ag85B specific T cells) of untreated and GM-CSF (2 ng/mL/2 × 10^5^ macrophages) treated human MDMs at day 1 and 3 post-infection. **(H)** Effect of exogenous addition of GM-CSF on cell viability of MTB-infected and uninfected human MDMs with time. Data represent the average of three independent experiments carried out in duplicate. Bars and error bars represent means and SD, respectively. *p ≤ 0.05, **p ≤ 0.005, ***p ≤ 0.0005, ****p ≤ 0.0001.

Interestingly, the viability decreased significantly, while the bacterial burden increased, with time in mouse MDMs (Figure 1C). In contrast, human MDMs exhibited less cell death after infection with MTB. Surprisingly, even uninfected mouse MDMs were unable to survive beyond 7 days as compared to human MDMs, which were able to maintain nearly 50% viability until day 21 (Figure 1D).

Because human MDMs produced more GM-CSF as compared to mouse MDMs, we hypothesized that GM-CSF may contribute to the increased survival of human MDMs. To test this hypothesis, we supplemented mouse GM-CSF to mouse MDMs cultivated *in vitro* and examined their viability before and after MTB infection. As expected, the addition of GM-CSF to mouse MDMs significantly increased the longevity of both uninfected and MTB-infected mouse MDMs (Figure 1E). The uninfected and MTB-infected mouse MDMs supplemented with 2 ng of mouse GM-CSF were able to maintain more than 60% cell viability until day 21. The overall bacterial burden in both mouse and human MDMs was significantly reduced with supplementary GM-CSF (Figure 1F).

We also observed that exogenous addition of GM-CSF enhanced secretion of IL-2 by antigen 85B specific T cells (F9A6) upon overlay to infected human MDMs, indicating a role for GM-CSF in improving antigen processing and presentation (Figure 1G). The GM-CSF-mediated increased antigen presentation by MTB*-*infected human MDMs became more prominent with time, indicating that GM-CSF gradually increased the fusion of bacteria-containing phagosomes with lysosomes. This could explain the observed decrease in bacterial load in the human cells (Figure 1F). Remarkably, human MDMs were also able to maintain their cell viability for a more extended period of time when GM-CSF was externally added to infected/uninfected cells (Figure 1H).

These results indicate that GM-CSF helped macrophage control MTB by decreasing bacterial load, while also preventing host cell death. These findings on human vs. murine monocyte-derived macrophages also indicate that the ability of macrophages to contain MTB infection could be due to the dual role of GM-CSF in prolonging the host cell survival as well as stimulating intracellular anti-MTB effector functions. Though not studied during MTB infection, GM-CSF has been known to modulate the developmental as well as effector functions of different lung macrophage populations (14). GM-CSF can stimulate oxidative metabolism, Fc-dependent phagocytosis, and expression of class II major histocompatibility complex to boost the effector functions of macrophages (Figure 2). Based on these evidence together, it is intriguing to investigate whether lung resident macrophages that produce GM-CSF or respond to GM-CSF signaling could influence the outcome of MTB infection.

**Figure 2:**
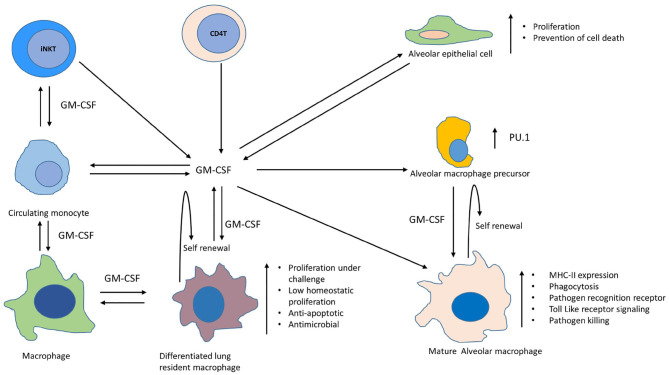
Involvement of GM-CSF in differentiation, self-renewal, proliferation, and expansion of antimicrobial functions of different macrophage populations within lungs. Depicted are macrophages, monocytes, alveolar macrophages, alveolar epithelial cells, and other non-myeloid cells that produce GM-CSF in lungs during homeostasis and infection. Maturation of alveolar macrophage occurs in presence of GM-CSF leading to augmentation of their antimicrobial functions, such as increased expression of MHC-II, pathogen recognition receptors, activation of toll like receptor signaling and enhanced pathogen killing (14). GM-CSF is also required for self-renewal of AMs (15). Transcription factor PU.1 mediates this GM-CSF-dependent effects on differentiation of AMs and their innate immune functions during infections (16). Circulating monocytes can also be recruited to lungs during infections and GM-CSF assists in their differentiation into macrophages (17–19). GM-CSF further helps these differentiated macrophage in maintaining their self-renewal and a low homeostatic proliferation in the lungs during health, whereas challenge with infection/injury/inflammation can induce their proliferation in a GM-CSF dependent manner. These fully differentiated macrophages also exhibit strong anti-apoptotic and antimicrobial properties. Alveolar epithelial cells also produce GM-CSF which not only helps in clearance of surfactant proteins and lipids but also supports the differentiation of alveolar and recruited macrophages along with their innate effector functions against infections within lungs (9, 10, 20, 21). GM-CSF can also be produced by CD4 T and iNK T cells which can further contribute to the optimum level of this cytokine required for sustained macrophage effector functions against TB pathogen (11, 22).

The role of GM-CSF is also well-defined in the self-renewal of macrophages, which is one of the mechanisms used to maintain a physiologically stable macrophage pool *in vivo* (23). Bone marrow-derived monocytes can settle in the lung during health as well as infection, and maintain their longevity through self-renewal (24, 25). Similarly, alveolar macrophages (AMs) also have the capacity of local self-renewal throughout life (15). GM-CSF is one of the critical intrinsic mitogenic signals required for self-renewal of both alveolar as well as bone marrow-derived macrophages in the lungs (Figure 2) (23). Macrophages of embryonic origin also require GM-CSF signaling to maintain the long-lived resident macrophage pool in the lungs. During the infection, increased production of GM-CSF has been shown to induce proliferation and differentiation of AMs which contributes to innate immunity in the lung (Figure 2) (16). Differentiation of AMs during infection is mediated through GM-CSF dependent increase in the expression of PU.1. Innate functions of AMs, such as cell adhesion, phagocytosis, pathogen killing, mannose-, and Toll-like receptor expression is promoted through PU.1. Macrophages derived from circulating monocytes also retain the capacity to proliferate and differentiate in the lungs during infection or inflammation and GM-CSF plays a pivotal role in the process (Figure 2) (17–19). While these fully differentiated macrophages can maintain a low homeostatic proliferation in the lungs during health, the challenge with infection/injury/inflammation can induce their proliferation strongly via GM-CSF. These differentiated lung macrophages also demonstrate increased antimicrobial properties. Though not studied in the context of MTB infection, evidences from earlier studies suggest that GM-CSF is essential to prevent apoptosis of macrophages during their differentiation (Figure 2) (26). We also observed a direct relationship between the levels of GM-CSF produced by macrophages and their ability to prevent cell death during MTB infection (Figures 1A,C,D). It is thus worth exploring whether this characteristic of self-renewal through GM-CSF helps against MTB infection *in vivo* as well. Simultaneous examination of these cellular processes during MTB infection can help us conclude whether GM-CSF signaling, cell differentiation, and cell death/survival pathways are linked with protective innate responses of macrophages. Because the lung is the primary site of MTB infection, and alveolar and resident macrophages are the primary host cells for the pathogen, relative levels of GM-CSF in the lung may determine macrophage function and their biological properties that, in turn, may influence the outcome of infection. Though in a murine model of TB, the protective role of GM-CSF producing non-myeloid cells, such as iNKT and CD4 T cells has also been reported during MTB infection which suggests that perhaps these cells could also be contributing to maintain an optimum level of GM-CSF required for the effective functioning of macrophages (Figure 2) (11, 22). While the local proliferation of alveolar and resident macrophages has been reported in humans as well as mice (27, 28), the latter is naturally more susceptible to TB. Infection of mice with MTB leads to progressive disease in all animals resulting in their premature death, in contrast to human populations that do not develop the primary disease in 90% cases. Considering this difference, how the levels and cellular source of GM-CSF in mice vs. human lungs differ before and after infection with MTB is important to understand. Multiple prolonged investigations so far have failed to find any consistent correlates of immunity that can distinguish adults who develop clinical TB from those who remain healthy. Most of these investigations have been carried out in experimental animal models that do not resemble the true immunopathological events that naturally occur during different outcomes in humans after exposure to MTB. In order to find the immune correlates and GM-CSF mediated mechanism of protection against TB, it is imperative to compare the immunopathological events that unfold in TB susceptible vs. TB resistant individuals/animal models after exposure to pathogen. While it is challenging to conduct such studies in humans, experimental animal models of rhesus macaque, cynomolgus macaque, and rabbit, have been developed in the past which could be explored to investigate the role of GM-CSF during different outcomes of MTB infection (29–31). It is also worth investigating if differences in GM-CSF levels and cellular sources within and outside of the lungs exist in TB-susceptible vs. TB-resistant human populations. The existence of polymorphisms in the GM-CSF gene or its receptor/s also needs to be analyzed to further validate the role of GM-CSF in innate immunity to TB. Answers to these unresolved questions through future studies may well envision a therapeutic role for GM-CSF, either through host-directed therapies or vaccines that elicit optimal GM-CSF production to control MTB.

## Data Availability Statement

All datasets generated for this study are included in the article/Supplementary Material.

## Ethics Statement

The studies involving human participants were reviewed and approved by UTHSC-Houston IRB. Written informed consent for participation was not required for this study in accordance with the national legislation and the institutional requirements.

## Author Contributions

AK designed the study. AM and VS led the project, conducted the experiments and analyzed the data. AK wrote the manuscript. SS edited the manuscript. CJ, JA, and RH provided the feedback on the manuscript.

## Conflict of Interest

The authors declare that the research was conducted in the absence of any commercial or financial relationships that could be construed as a potential conflict of interest.
