# Supplementary material for: GM-CSF Dependent Differential Control of Mycobacterium tuberculosis Infection in Human and Mouse Macrophages: Is Macrophage Source of GM-CSF Critical to Tuberculosis Immunity?
Source: Front Immunol. 2020 Jul 23;11:1599. doi: 10.3389/fimmu.2020.01599 (PMC7390890; doi:10.3389/fimmu.2020.01599)
Supplement: Supplementary file 1 [file Data_Sheet_1.docx]

**Materials and Methods**

**Ethics statement**

All methods using human and animal cells were carried out in accordance with relevant guidelines and regulations from the Institutional Review Board of the University of Texas Health Science Center-Houston. All experimental protocols were approved by the University of Texas Institutional Biosafety Committee (UTHSC-IBC), which sanctioned the study (HSC-MS-15-0548). Healthy donor-peripheral blood monocytes were obtained from commercial sources and de-identified subjects; thus, no informed consent was necessary for their use in the *in vitro* cell culture studies, per the institutional human subjects review committee.

**Bacterial culture**

*M. tuberculosis* H37Rv (ATCC 27294) was obtained from ATCC and grown in Middlebrook 7H9 broth (Difco, Becton Dickinson) supplemented with 0.05% (v/v) Tween 80 and Middlebrook AODC Enrichment (Becton Dickinson) to mid-log phase (OD 600 nm = 0.6-0.8) and reseeded in fresh media 7 days before infection, as previously described ^1^ . Before using for monocyte derived macrophages (MDMs) infection, bacterial cultures were washed three times in PBS and sonicated at 4 watts for 60 seconds using a sonicator (60 Sonic Dismembrator, Fisher Scientific) to prepare a uniform single-cell suspension of bacteria.

**Isolation and cultivation of human and mouse MDMs**

Human MDMs were isolated from peripheral blood of healthy donors, and mouse MDMs were isolated from bone marrow. Cells were isolated and cultivated using density centrifugation and plastic adherence, and characterized by flow cytometry according to protocols previously described ^2^. More than 90 of adherent cells were monocytes as determined by CD14 (human) and CD11b (mouse) expression through flow cytometry (BD Biosciences). Monocytes were grown in Iscove’s Dulbecco Modified Medium (IMDM) with 10% FBS for 6 days and harvested with 0.05% trypsin/1 mM EDTA treatment (Life Technologies) for 5 min at 37°C and re-suspended with fresh culture medium (IMDM + 10% fetal bovine serum) for subsequent experiments in 6-, 24,- or 96-well plates or 8-chamber slides.

**Infection, cell viability, and Colony Forming Unit assays**

The bacterial suspension was diluted in serum-free medium for addition to MDMs at MOI 1:1 in 6-, 24-, or 96-well plates. After 4 hours of incubation, the media was replaced with fresh IMDM media containing 10% FBS. For long-term infections, the media was replaced on alternating days. Alamar Blue cell viability reagent (Life Technologies, DAL1025) was used to assess cell viability by adding the ready-to-use 1X solution to uninfected or *M. tuberculosis*-infected MDMs at various time points, followed by fluorescence measurement through fluorimeter per the manufacturer’s protocol. Intracellular bacterial counts at various times post-infection were determined by CFU assay on 7H11 agar plates. Uninfected controls were included for all time points.

**Antigen presentation and cytokine assays**

We have described in detail our antigen presentation assay ^3^, and the original method described by Harding et al. has been extensively used for *in vitro* antigen presentation from macrophages and dendritic cells ^4^. Briefly, *M. tuberculosis*-infected human MDMs were washed after a 4-hour infection and overlaid with the F9A6-CD4 T cell hybridoma (gifted by David Canaday), which recognizes an Ag85B epitope in the context of human HLA-DR1 ^5^. IL-2 secreted from hybridoma T cells, GM-CSF secreted from *M. tuberculosis* infected human, and mouse MDMs were determined using sandwich ELISA kits (Biolegend #432001, #432201, #431001) of respective cytokines. TNF-α, IL-10, IL-4 and IL-12 secreted by mouse and human MDMs were determined by collecting cell culture supernatants and titrating these cytokine levels through their respective sandwich ELISA kits (Biolegend #430901, #430201, #431101, #430301, #431411, #430601, #431601, #430701), following manufacturer’s instructions.

**Statistics**

For all statistical analyses, PRISM (Version 5, GraphPad, San Diego) software was used. Statistical analysis was performed with unpaired two-tailed Student’s t-test and one-way ANOVA with posthoc analysis. P values <0.05 were considered significant.

**References**

1. Chanwong, S., Maneekarn, N., Makonkawkeyoon, L. & Makonkawkeyoon, S. Intracellular growth and drug susceptibility of Mycobacterium tuberculosis in macrophages. *Tuberculosis (Edinb).* **87**, 130–3 (2007).

2. Sharma, M. *et al.* Intracellular survival of Mycobacterium tuberculosis in macrophages is modulated by phenotype of the pathogen and immune status of the host. *Int. J. mycobacteriology* **1**, 65–74 (2012).

3. Singh, C. R., Bakhru, P., Khan, A., Li, Q. B. & Jagannath, C. Cutting edge: Nicastrin and related components of γ-secretase generate a peptide epitope facilitating immune recognition of intracellular mycobacteria, through MHC class II-dependent priming of T cells. *J. Immunol.* **187**, (2011).

4. Gehring, A. J. *et al.* The Mycobacterium tuberculosis 19-Kilodalton Lipoprotein Inhibits Gamma Interferon-Regulated HLA-DR and Fc R1 on Human Macrophages through Toll-Like Receptor 2. *Infect. Immun.* **71**, 4487–4497 (2003).

5. Canaday, D. H. *et al.* T-cell hybridomas from HLA-transgenic mice as tools for analysis of human antigen processing. *J. Immunol. Methods* **281**, 129–42 (2003).
