# Supplementary material for: GM-CSF Dependent Differential Control of Mycobacterium tuberculosis Infection in Human and Mouse Macrophages: Is Macrophage Source of GM-CSF Critical to Tuberculosis Immunity?
Source: Front Immunol. 2020 Jul 23;11:1599. doi: 10.3389/fimmu.2020.01599 (PMC7390890; doi:10.3389/fimmu.2020.01599)
Supplement: Supplementary file 2 [file Data_Sheet_2.docx]

Supplementary figures

**Figure S1**

Figure S1: Time dependent GM-CSF production by human and mouse MDMs with and without LPS activation. Mouse and Human MDMs were cultivated in vitro following identical methods as described for Figure 1A. Results are the means ± SD of triplicate samples and are representative of three independent experiments. Bars and error bars represent means and SD, respectively. * = p value ≤ 0.05, ** = p value ≤ 0.005, *** = p value ≤ 0.0005, **** = p value ≤ 0.0001.

**Figure S2**

Figure S2: Cytokines secreted by human and mouse MDMs with and without MTB infection. Supernatants after 72 hours post infection/cultivation were collected and titrated with sandwich ELISA kits of each cytokine of their respective species. Results are the means ± SD of triplicate samples and are representative of three independent experiments. Bars and error bars represent means and SD, respectively. * = p value ≤ 0.05, ** = p value ≤ 0.005, *** = p value ≤ 0.0005, **** = p value ≤ 0.0001.
